# Development and Evaluation of Feline Tailored Amlodipine Besylate Mini-Tablets Using l-lysine as a Candidate Flavouring Agent

**DOI:** 10.3390/pharmaceutics12100917

**Published:** 2020-09-24

**Authors:** Chinedu S. Ekweremadu, Hend E. Abdelhakim, Duncan Q. M. Craig, Susan A. Barker

**Affiliations:** 1 Department of Pharmaceutics, University College London School of Pharmacy, 29-39 Brunswick Square, London WC1N 1AX, UK; chinedu.ekweremadu.17@alumni.ucl.ac.uk (C.S.E.); hend.abdelhakim.16@ucl.ac.uk (H.E.A.); duncan.craig@ucl.ac.uk (D.Q.M.C.); 2 Medway School of Pharmacy, The Universities of Greenwich and Kent at Medway, Chatham, Kent ME4 4TB, UK

**Keywords:** mini-tablets, amlodipine, taste, feline, l-lysine, E-tongue, hypertension

## Abstract

Felines may find orally administered medicines unpalatable, thus presenting a problem in the treatment of chronic conditions such as hypertension, a commonly diagnosed condition in felines requiring daily administration of medication. A pertinent example is amlodipine besylate, formulations of which are known to be poorly tolerated by cats. There is therefore a need to develop feline-specific delivery approaches that are both simple to administer and mask the taste of the drug, thereby enhancing the owner’s commitment to treatment and the associated therapeutic outcome for the companion animal. In addition, it is helpful to develop accessible and reproducible means of assessing taste for pre-clinical selection, hence the use of recently developed taste biosensor systems for veterinary applications is an area of interest. This study focuses on developing feline-specific amlodipine besylate formulations by improving the taste using a suitable flavouring agent while reducing dosage form size to a 2 mm diameter mini-tablet. The choice of l-lysine as a flavouring agent was based on the dietary and taste preference of cats. The impact of l-lysine on the taste perception of the formulation was evaluated using a biosensor system (E-tongue) fitted with sensors sensitive to bitter tastes. The results showed l-lysine successfully masked bitterness, while the drug release studies suggest that it has no impact on drug dissolution. In addition, tableting parameters such as tablet mass uniformity, content uniformity, tablet diameter, thickness and hardness were all satisfactory. The present study suggests that amlodipine besylate mini-tablets containing l-lysine could improve the palatability and in turn support product acceptability and ease of administration. These data could have an impact on orally administered medicines for cats and other veterinary species through product differentiation and competitive advantage in the companion animal market sector. The study also outlines the use of the electronic tongue as a tool for formulation selection in the veterinary field.

## 1. Introduction

Oral administration is particularly challenging in felines; cats are considered to be less compliant than other companion animal species, thereby reducing voluntary tablet acceptance and administration [1]. Forcing a cat to take medication can lead to owner injuries and also have a negative consequence on the cat-owner relationship [2]. This poses a significant problem, especially in the treatment of chronic diseases that affect felines, such as hypertension, where daily administration of medicine is required. In such cases, the pet owner’s commitment to administering the course of treatment is crucial. The challenges of oral administration of medicine in cats may be attributed to the unpalatable nature of the currently available perioral dosage forms [3]. Cats almost invariably do not willingly take tablets, with the problem being compounded if the taste is unpleasant. In order to avoid these drawbacks, the solid dosage form is sometimes hidden in a more palatable material, such as food, by pet owners [4]. Some limitations to this approach include the fact that some pharmaceuticals need to be administered in the fasted state [5]. In addition, the active drug may be so bitter that the palatable food will not mask it successfully [4], or else the animals will often eat the food around the medicine and leave the dosage form [1]. Interactions with palatable substances may also be an issue; putting tetracycline in milk, for example, leads to the formation of tetracycline-metal complexes which are either insoluble or poorly absorbable from the gastro-intestinal tract [6].

Due to the limitations of concealing medicines in palatable food, feline-specific medicines are needed so that cats are more likely to voluntarily accept medicines [4]; this would increase medication adherence and improve the success of the treatment [7]. In addition, product differentiation through palatable formulations is an important means of gaining a competitive edge in the companion animal segment market [3]. In the case of feline hypertension, cats not adherent to their medication have the highest risk of developing complications such as retinal detachment, neurological disorders, congestive heart failure and kidney failure [8].

Amlodipine besylate (AB) is the drug of choice used for the management of feline hypertension, although the currently available formulations have drawbacks. AB is a long-acting calcium channel blocker prescribed by veterinarians for the management of hypertension, especially in cats with kidney disease [9]. The commercially available human AB tablet (5 mg or 10 mg) has to be scored into fractions to dose cats. From the pet owner’s point of view, the scoring of tablets could decrease treatment commitment and lead to discontinuation of therapy, while scoring the tablet into fractions could lead to inaccuracy of dosing. Another drawback of the use of human AB tablets in cats is the bitter taste which felines find unappealing.

In order to improve drug compliance through formulating oral palatable formulations, there is a need to understand the taste and dietary preference of cats so as to formulate medicines which they would find palatable. Cats are strict carnivores and, as a result, have a preference for meat-based diets and umami flavour [10,11]. On that basis, Amodip^®^ (Amlodipine 1.25 mg), a chicken-flavoured chewable tablet, has been licensed for use in cats [12]. Other feline specific formulations include Heartgard^®^ (ivermectin), a beef-based chewable tablet for the treatment of gastrointestinal parasites in cats, which has been commercialized and has shown good palatability [13]. AthriCare^TM^ is chewable and is made from a roast beef and liver flavour-containing base, and has been marketed for use in veterinary practice [4]. However, a potential drawback of flavours of natural origin is they contain multiple components, leading to stability issues as these components could interact with the API and alter dissolution and/or absorption [1]. In addition, the manufacturing process is more complicated, and microbial contamination, as well as batch-to-batch variation of the formulation’s flavour, is a problem. Meat-based flavours pose additional risks due to bovine spongiform encephalopathy [4]. As a result, there is a need to replace these natural flavours with synthetic and one-component flavours appealing to cats, such as amino acids. The amino acid l-lysine was chosen for this study because of the carnivorous nature of cats and their preference for umami flavour, as this and other amino acids are meat precursors [14]. l-lysine is one of the nine essential amino acids; it has a role as a micronutrient and a nutraceutical, and also provides flavour [15]. In addition, l-lysine undergoes Strecker degradation to the aldehyde form, which may also make a contribution to flavour [16]. No safety concerns have yet been associated with this amino acid when used as a flavouring agent, thus making this material a good candidate for the present study. Previous studies on cats have indicated positive outcomes when using l-lysine [10,17,18]; however, it should be noted that a behavioural study by Savolainen et al. (2019) found that felines did not find the amino acids tested in their study (which did not include l-lysine) especially palatable [2], hence there remains some uncertainty regarding the applicability of the approach.

**Figure 1:**
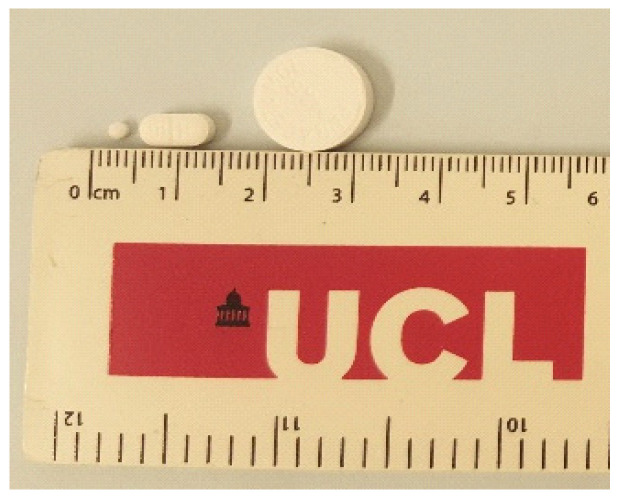
Size of a typical mini-tablet (left) compared to conventional tablets.

As is the case for children, conventionally sized tablets are not suitable for pets due to swallowing difficulties [19]. Studies in paediatrics have shown that mini-tablets are well accepted and easy to swallow formulations; 3 mm in diameter mini-tablets are easily swallowed by children aged two to six [20] (Figure 1). In another study, children aged six months to one year were able to swallow 2 mm in diameter tablets [21]. Studies by Savolainenet al. [2] in cats indicated that mini-tablets may not necessarily be acceptable alone, but were palatable when placed inside a food item. For this reason, mini-tablets were considered in this present study because of their potential to resolve swallowing difficulties due to their small size.

Taste assessment is a vital quality-control parameter for evaluating taste-masked formulations [22]. In the past, panels of human volunteers and laboratory animals, specifically mice and rats, were the only methods to determine the taste of formulations, but the limitation of these approaches are the ethical considerations, potential toxicity of medicines, the cost and time involved, and finally the fact that results from human panelists vary depending on the sex, age, eating habits and origin of the taster [23]. Additionally, transferring the results of human taste panels to the taste impression of felines could be accompanied with significant falsification, as their taste impression varies between species, particularly in the spectra of compounds to which each taste group corresponds. For example, unlike humans and most mammals, cats are unable to detect the sweetness of sugar and high intensity sweeteners due to their lack of receptors for the detection of sweet stimuli [24].

In order to address these limitations, the assessment of taste is required, and to this end there is considerable interest in the use of electronic tasting systems, or E-tongue technology [25,26]. The E-tongue is an analytical tool that provides the assessment of taste by means of particular sensor membranes and electrochemical techniques [25]. E-tongues attempt to represent and imitate the interaction of molecules with biological taste buds [27]. In this study, the E-tongue was employed to assess the effectiveness of l-lysine as a taste-masking agent.

The primary aim of this study was to improve the palatability and ease of administration of amlodipine besylate via the development of mini-tablets containing the flavouring agent l-lysine, while the taste has been evaluated using the electronic tongue with a particular view to identifying the taste-masking properties of l-lysine and exploring the potential of this approach for veterinary applications.

## 2. Materials and Methods

### 2.1. Materials

Amlodipine besylate (AB) was purchased from LKT Laboratories (St Paul, MN, USA). The proposed formulation contained the following excipients: partially pregelatinized maize starch (Starch 1500, Colorcon, Kent, UK), Disintequik MCC 25 (Foremost Farms, Baraboo, WI, USA), l-lysine monohydrochloride (Scientific Laboratories Suppliers, Nottingham, UK). Magnesium stearate, quinine hydrochloride dihydrate, potassium chloride, tartaric acid, potassium hydroxide, absolute ethanol and hydrochloric acid (32%) were purchased from Sigma-Aldrich (Dorset, UK).

### 2.2. Compression of Mini-Tablets

Four formulations were prepared; the composition of each formulation is listed in Table 1. The mini-tablets had a target diameter of 2 mm and weight of 6.5 mg which contained 1 mg of AB and were prepared by direct compression. All excipients used were individually sieved to obtain a particle size of 125 to 250 µm, and then mixed using a rotatory mixer (Pascal Engineering, Hatfield, UK) for 14 min. Magnesium stearate was added last and then mixing was continued for an additional 10 min. In order to evaluate manufacturing parameters for the direct compression, different compression force and tableting speed values were tested using a Piccola tablet press (Riva Europe, Shropshire, UK) fitted with 2 mm round concave punches applying compression forces in the range of 0.65 to 1.59 kN and a tableting speed of 10 RPM. Placebo mini-tablets were prepared using a similar procedure.

### 2.3. Characterisation of Mini-Tablets

#### 2.3.1. Mass Uniformity, Thickness and Diameter

In total, 20 mini-tablets were randomly selected and individually weighed using XPE Analytical balance (Mettler Toledo, Leicester, UK). The diameter and thickness of the tablets were determined using a digital caliper (Mitutoyo model Pk-0505, Mitutoyo, Kawasaki, Japan). The mean values and the relative standard deviation were calculated.

#### 2.3.2. Tablet Strength (Hardness)

In total, 20 randomly selected mini-tablets were tested to check the maximum force needed to break the tablet by using a Texture Analyser TA.XT Plus (Stable Micro Systems, Surrey, UK) following the method outlined by Choonara et al. (2006) [28].

#### 2.3.3. Content Uniformity

In total, 10 randomly selected tablets were crushed into fine powder and dissolved in 10 mL of methanol. The flask contents were shaken throughout, then 3 mL of filtered samples were withdrawn and analysed spectrophotometrically at wavelength 362 nm using a Jenway 6305 spectrophotometer (Jenway, Stone, UK). The AB content was calculated on the basis of the calibration curve of AB in methanol (linearity in the range of 20–160 µg/mL, *R*^2^ = 0.9985). Data collection and analysis were conducted with Microsoft Excel 2016 software. The studies were carried out in triplicate [29].

#### 2.3.4. In-Vitro Dissolution Test

Dissolution of the AB tablet was performed under sink conditions using a shake incubator (SciQuip, Newtown, UK) and the rotational speed was set at 50 RPM [30]. The dissolution medium was 50 mL of 0.016 N HCl (pH 1.8) to represent the pH of the cat stomach with chronic kidney disease (a condition associated with feline hypertension [31]). The temperature of dissolution was maintained at 38.5 ± 0.5 °C throughout the experiment to represent the temperature found in-vivo.

Quantities of 3 mL of sample were withdrawn at specific time intervals and replaced by fresh 3 mL quantities of HCl. The amount of drug released was detected using a UV-Jenway 6305 spectrophotometer (Jenway, Stone, UK), and samples were analysed at a wavelength of 362 nm, which is an experimental λ_max_. The AB content was calculated on the basis of calibration curve of AB in HCl (linearity in the range of 20–160 µg/mL, *R*^2^ = 0.9994). Each formulation was analysed in triplicate.

### 2.4. Electronic Taste Sensing System Measurement

#### 2.4.1. Dose Response Curve

A dose response curve of AB in 10 mM KCl was prepared; the quinine hydrochloride dihydrate dose response was performed over the same concentration range as AB so as to enable comparisons and assess the bitterness threshold of AB. The recommended medium for E-tongue use is 10 mM KCl. 

#### 2.4.2. Sample Preparation

For the four different batches of mini-tablets, 20 tablets were randomly selected, milled separately in a mortar and placed in 100 mL of 10 mM KCl at 37 °C for 1 min followed by filtration using 0.22 µm filters (Merck-Millipore, Cork, Ireland). The concentration of the sample of AB mini-tablet prepared was 1 mg/5 mL or 0.353 mM, to mimic the clinical dose in an approximate saliva volume of 5 mL. The physical mixture of excipients and individual excipients used in the formulation were also assessed, by dissolving the equivalent amount in 20 tablets in 100 mL of 10 mM KCl at 37 °C for 1 min followed by filtration using 0.22 µm filters (Merck-Millipore, Cork, Ireland). The composition of the physical mixture was AB, magnesium stearate, starch and Disintiquick and the proportions were equivalent to those found in Formulation A. 

#### 2.4.3. Sample Measurement

The TS-5000Z taste sensor (Intelligent Sensor Technology Inc., Atsugi, Japan) was used. The sensors BT0 (detects basic bitterness), AC0 (developed to detect to bitter cationic substances), AN0 (bitter cationic and neutral substances), C00 (acidic bitterness) and AE1 (astringency) were used to determine the bitterness intensities of AB, quinine HCl dihydrate and the sample solutions prepared. Sensor checks were carried out before every measurement to ensure the sensors were working in the correct mV range. Each sample was measured four times. The first run was discarded as recommended by the supplier to allow for sensor conditioning. The reference solution was prepared by dissolving 30 mM potassium chloride and 0.3 mM tartaric acid in distilled water. The negatively charged membrane washing solution was prepared by diluting absolute ethanol to 30% *v*/*v* with distilled water, followed by the addition of 100 mM hydrochloric acid. The positively charged membrane washing solution was prepared by diluting absolute ethanol to 30% *v*/*v* and adding 100 mM potassium chloride and 10 mM potassium hydroxide to the mixture. All substances were used as received. This method is adapted from Abdelhakim et al. (2019) [32].

Taste sensor output is obtained by measuring the difference in electric potential between the taste sensor and the reference electrode. Dose response curves for AB and quinine HCl dihydrate were generated by testing those drugs at concentrations ranging between 0.01 mM and 10 mM, corresponding to concentrations equivalent to intervals on the logarithmic scale.

Each measurement cycle consisted of the following elements:

1. Measurement of reference potential (V_r_) in reference solution for 30 s;
2. Measurement of electric potential (V_s_) in sample (initial taste) for 30 s;
3. Lightly washing of sensors in reference solution;
4. Measurement of electric potential (V_r1_) in reference solution again (aftertaste or CPA) for 30 s;
5. Refreshing of sensors in alcohol solution to give them a complete wash before the measurement of the next sample for 30 s.

The initial and aftertaste were derived via the following: V_s_ − V_r_ = initial taste and V_r1_ − V_r_ = aftertaste.

### 2.5. Statistical Analysis

All data analysis were carried out using OriginPro 9.4 (Origin Lab, Northampton, MA, USA).

## 3. Results

### 3.1. Physical Characterisation of Mini-Tablets

The mini-tablets prepared by direct compression had satisfactory appearances, and the results of their physical characterisation are presented in Table 2.

All mini-tablet formulation batches were within the limits of the European pharmacopeia specifications for weight variability of less than 10% from the mean of individual samples. All batches passed the test of the uniformity of weight according to the guidelines stated in the Ph. Eur. monograph 2.9.5 for the uniformity of mass of single dose preparations [33]. This may be attributed to the excellent flowability of the powders and narrow maximum particle size (125–250 µm). This reduces the dwell time of the powders in the feed and avoids the formation of void space in the tablet, thereby ensuring even powder filling [33]. The mini-tablets also had consistent thicknesses and diameters, although there was a greater variation in the hardness values. This parameter is of particular significance for mini-tablets, as there is the additional challenge compared to conventional systems of producing tablets with sufficient robustness without damaging the punches; to this effect, Disintequik (co-processed alpha lactose monohydrate and microcrystalline cellulose) was selected due to it providing high mechanical strength under direct compression. Examination of Table 2 indicates that tablets containing the flavouring l-lysine had lower mechanical strength; this may be a result of the presence of the flavouring or the lower proportion of bulking agent, or a combination of the two.

### 3.2. Electronic Taste Sensing System Measurement

#### 3.2.1. AB Taste Assessment

**Figure 2:**
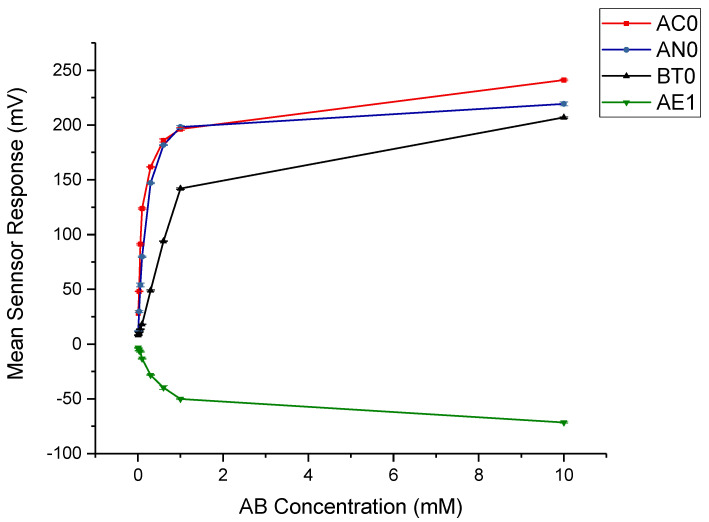
Dose response curve representing initial taste for amlodipine besylate as tested by AC0, AN0, BT0 and AE1.

The taste sensing system was first used to quantify the sensor response for AB as a function of its concentration, as seen in Figure 2. AC0, AN0 and BT0 are negative taste sensors and stay in the negative range if they do not detect bitter substances. Anything under 5 mV is considered not detectable by these sensors, or not bitter. As seen in Figure 2, AB is strongly detected by all three sensors, indicating a highly basic bitter drug profile. Out of the three sensors, AC0 represents the sensor most suitable for detecting AB, as it showed the highest sensitivity of detection for initial taste. AE1 is a positive taste sensor which represents astringency, and as seen in Figure 2, AB demonstrates some degree of astringency.

The positive sensor C00 was also used to test AB’s bitterness, however a negligible response was recorded which indicates no acidic bitterness was detected, which is expected for a drug with a basic character.

**Figure 3:**
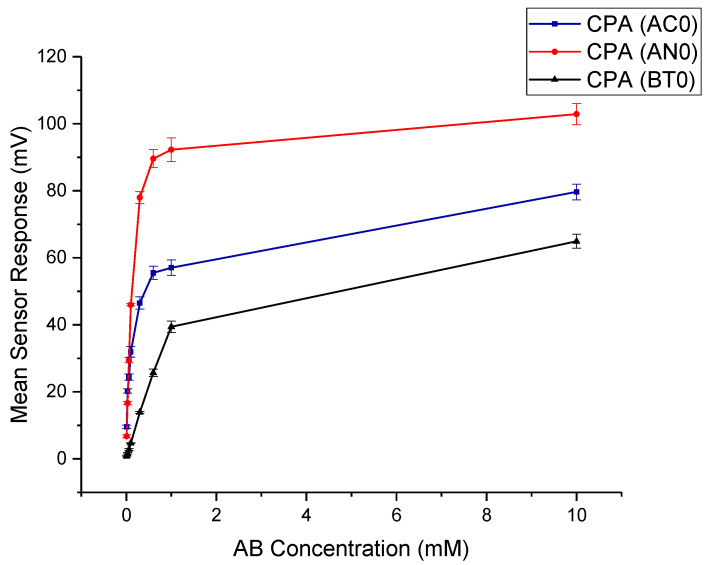
Dose response curve representing aftertaste of amlodipine besylate as tested by AC0, AN0 and BT0.

Aftertaste or CPA is the most reliable way to display bitterness data, as bitterness manifests itself through the adsorption of drug molecules on the taste sensor, or the human tongue. Figure 3 shows the aftertaste profile of the drug as detected by the basic bitterness taste sensors. AC0, AN0 and BT0 all detected a response for aftertaste bitterness, which confirms the need for a taste-masking strategy for AB. For astringency, sensor AE1 did not detect a response higher than 5 mV, which indicates that AB did not exhibit long-lasting astringency that manifests itself as an aftertaste, and therefore is not expected to cause an aversiveness challenge.

#### 3.2.2. AB Bitterness Threshold as Compared to Quinine Hydrochloride Dihydrate

**Figure 4:**
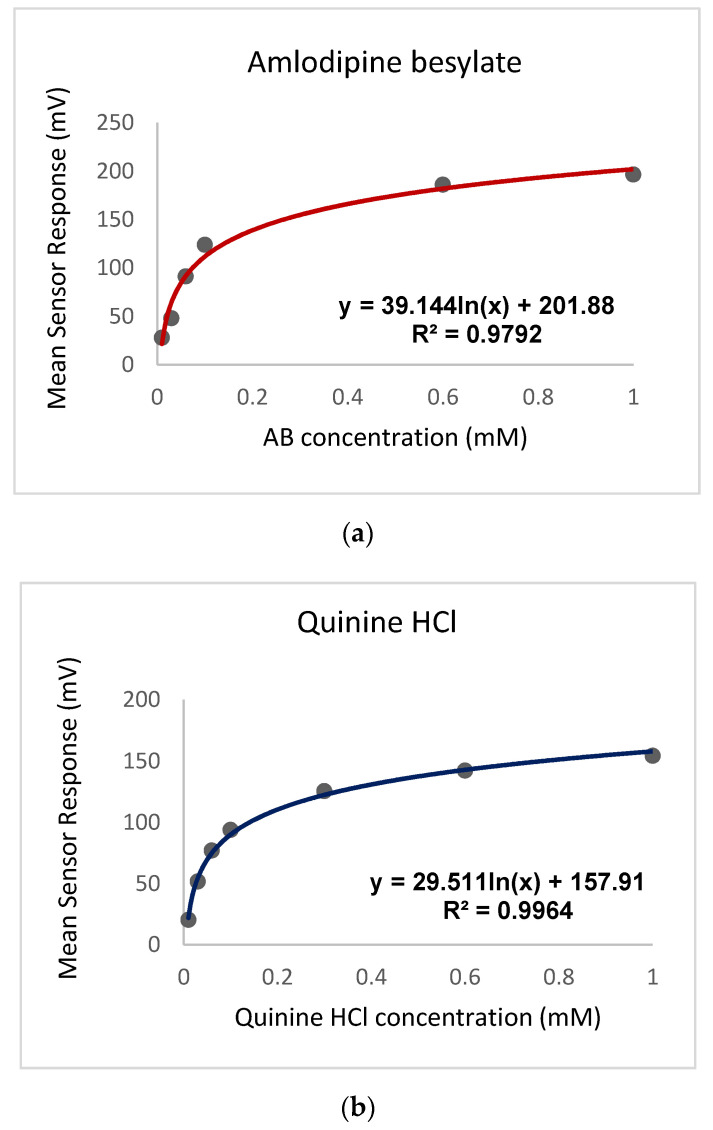
AC0 logarithmic trend-line sensor response curve showing initial taste for (**a**) amlodipine besylate and (**b**) quinine HCl dihydrate.

Quinine hydrochloride dihydrate was chosen as a reference bitter drug [34], and the bitterness profile using the AC0 sensor output for initial taste is shown in comparison to AB in Figure 4. Studies by Soto et al. (2018) [35] determined the EC_50_ bitterness threshold of quinine from human sensory panels to be 0.26 mM, this being the concentration corresponding to half of the maximum taste rating. This equates to an E-tongue AC0 sensor output of 118 mV when fitted on a logarithmic trend-line using the equation shown in Figure 4. 

To estimate the bitterness threshold of AB, this sensor value was used and substituted into the corresponding logarithmic equation to generate a mean drug concentration that matches quinine’s known sensor response corresponding to the EC_50_ (118 mV). The bitterness threshold of AB can therefore be assumed to be 0.2 mM, as calculated using the equation in Figure 4a. This indicates that the drug is more bitter than quinine hydrochloride dihydrate, thus clearly indicating a need for the AB to be taste-masked.

#### 3.2.3. Aftertaste of AB

Given that aftertaste, measured as the change in membrane potential caused by adsorption (CPA), is considered to be a useful measurement of bitter taste, this value was used to compare the formulations and raw materials.

**Figure 5:**
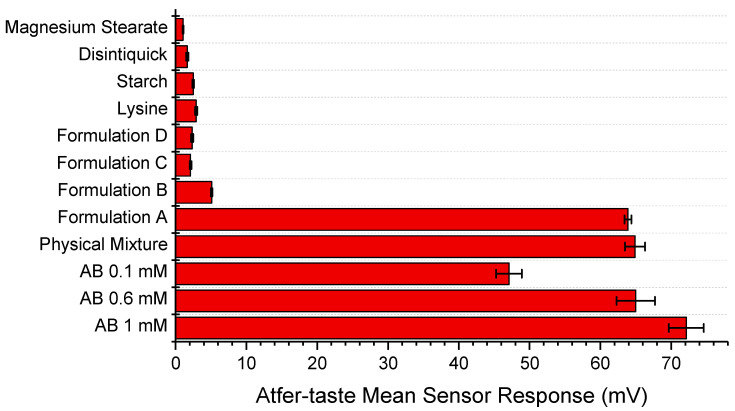
A chart comparing the aftertaste or CPA measurements (AC0 sensor) of the various excipients, the physical mixture without l-lysine and the formulated AB mini-tablets, as measured by the AC0 sensor.

Figure 5 shows a chart with the different CPA values of all the excipients, active pharmaceutical ingredients and formulations. The physical mixture contained all the excipients including the drug, but did not contain the masking agent l-lysine (hence being equivalent to Formulation A in Table 1). This is to show if any of the other excipients had a taste-masking function themselves, which they do somewhat appear to, compared to AB on its own at all concentrations. The mini-tablets with AB, with or without the taste-masking agent l-lysine, both contained the drug at a concentration of 0.353 mM or 1 mg/5 mL in the test solution.

Amongst all formulated mini-tablets, Formulation A exhibits the highest bitterness amongst the formulated products as it was non-masked, whereas Formulation B, which contained the drug and l-lysine, showed very low bitterness, demonstrating successful taste-masking. It is to be noted that any response under 5 kV is considered non detectable by the E-tongue and therefore non bitter, as seen with all the excipients, and Formulations C and D.

Comparison between the drug-containing mini-tablets suggests that Formulation A, which had no l-lysine, was considerably more bitter than Formulation B, which contained l-lysine. This difference shows that l-lysine has an impact on the taste-masking of AB when formulated into a mini-tablet, as shown in Figure 5.

### 3.3. Content Uniformity and In-Vitro Dissolution Studies

**Figure 6:**
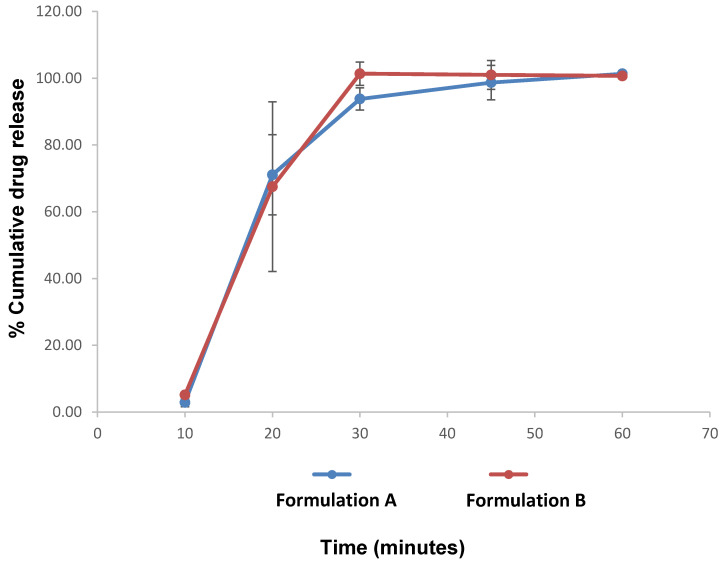
Dissolution profile of Formulation A (AB non-masked mini-tablets) and Formulation B (AB masked mini-tablets).

The drug contents of both API formulations comply with the criteria stipulated by the Ph. Eur. Method 2.9.6. The outcome of the drug release studies indicate that both l-lysine containing AB mini-tablets and the l-lysine-devoid AB formulation were immediate-release formulations, as more than 80% of drug release occurred within the first 45 min, as stipulated by Ph. Eur. (5.17.1). Additionally, they both had similar drug release profiles, as depicted in Figure 6. 

The in-vitro dissolution studies were performed at pH 1.8 and at 38.5 °C so that the gastric pH conditions and temperature of cats were mimicked [31]. The in-vitro drug release studies of Formulations A and B depicted in Figure 6 showed insignificant drug release within the first 10 minutes, which means drug release may not occur when the formulation is in the mouth unless chewed by the cat; the small size of the tablet may mitigate against this occurring. The delay in the onset of dissolution could be attributed to the slow disintegration due to the high level of hardness, in turn due to the choice of bulking agent (Disintequik MCC-25). Overall, however, the data indicates that the presence of the l-lysine did not appear to markedly influence the release profile of the drug, although the formulation containing the amino acid (Formulation B) did reach complete release slightly faster than the non-masked equivalent, possibly reflecting the lower hardness for the former tablets.

## 4. Discussion

The first purpose of the study was to investigate the dual approach of using mini-tablets as well as the incorporation of the taste-masking agent l-lysine to improve the palatability of the anti-hypertensive drug amlodipine besylate for administration to cats. The study showed that it is possible to produce satisfactory mini-tablets containing AB using direct compression, with the judicious choice of excipients allowing tablets of sufficient mechanical strength to be manufactured. This is an encouraging result, as the production of mini-tablets does present challenges not seen for larger tablets, although it was noted that there was a lag in initial dissolution, which may be associated with the mechanical strength. The presence of the masking agent, l-lysine, did result in some decrease in mechanical strength, although whether this is a reflection of the compression properties of the amino-acid, the alteration of the proportions of the other excipients, or both, is not clear at this point. Nevertheless, the overarching finding is that it is indeed possible to produce mini-tablets containing the drug and masking agent.

The second purpose was to explore the use of the electronic tongue as a means of assessing formulations for veterinary applications. The data indicated that the presence of l-lysine produces a marked reduction in the bitterness levels of AB. The raw materials themselves, other than the drug, showed low levels of bitterness, while the AB showed a markedly higher profile, this facilitating assessment of whether masking was occurring. It was also noted that the results for the AB alone, the non-masked tablets and the corresponding physical mixtures were all in broad agreement. The reduction seen for the masked formulations was very marked, and can therefore be considered to be a very encouraging result. These findings are consistent with research showing that amino acids have flavouring and taste-masking capabilities [16]. The findings in this present study are also in line with previous neurophysiological findings on the effect of amino acids on anesthetised cats [18], and are consistent with palatability studies using amino acids carried out on laboratory cats [10,17]. 

The limitations of the study must, however, also be considered. The bitter E-tongue sensor was used instead of an umami E-tongue sensor; as l-lysine is an umami flavour which is favoured by cats. A further study using this sensor could indicate acceptability rather than simply a lack of aversion (although the latter was the main point of the current study). More importantly, in order to confirm these findings, in-vivo studies are required, more specifically in the form of behavioural palatability studies stipulated in EMA/CVMP/EWP/206024/2011 guideline on the demonstration of palatability of veterinary medicinal products, in order to be able market the product as palatable [36]. The viability of this approach as compared to the established, chewable product (Amodip^®^) would also be of interest, not least because the approach outlined here is potentially applicable to a range of feline medications, and hence the comparative efficacy of the different available approaches would be of interest within the field. Finally, it would be of interest to take forward the electronic tongue studies to establish the relationship between the biosensor profile and in-vivo feline palatability. 

## 5. Conclusions

The administration of medicines to cats may be highly challenging, compounded by problems of taste if the therapeutic agent is bitter. The present study indicates that the mini-tablets containing amlodipine besylate and the taste-masking amino acid l-lysine may be successfully prepared by direct compression, with satisfactory release profiles being observed irrespective of the presence of the masking agent. Studies using the electronic tongue fitted with bitterness sensors indicate a marked decrease in aftertaste bitterness in the presence of l-lysine, while AB alone, non-masked formulations and physical mixtures all showed a strong response; indeed, one of the findings of the study is that AB is more bitter than quinine HCl, demonstrating the extent of the problem with this drug. The investigation is therefore highly encouraging in terms of both promoting the use of mini-tablets for feline delivery and the incorporation of l-lysine as a masking agent, although in-vivo studies will be required in order to confirm these findings.

## Figures and Tables

**Table 1:** Composition of mini-tablets containing Amlodipine Besylate (% *w*/*w*).

		Mini-Tablet Composition (% *w*/*w*)			
Ingredients	Functions	Formulation A (AB Unmasked)	Formulation B (AB Masked)	Formulation C (Placebo Unmasked)	Formulation D (Placebo Masked)
AB	API	15.4	15.4	0	0
l-lysine	Taste-masking agent	0	10	0	10
Starch-1500	Disintegrant	10	10	10	10
Mg Stearate	Lubricant	2	2	2	2
Disintequik	Bulking agent	72.6	62.6	88	78

**Table 2:** Physical characterisation results for the different formulations.

Formulation	Weight Variation (mg) ± SD	Thickness (mm) ± SD	Diameter (mm) ± SD	Hardness (N) ± SD	Content Uniformity (%) ± SD
A	6.26 ± 0.20	1.59 ± 0.08	1.98 ± 0.02	10.80 ± 2.20	103 ± 5.00
B	6.29 ± 0.30	1.66 ± 0.05	1.98 ± 0.02	8.83 ± 0.40	105 ± 1.40
C	6.39 ± 0.20	1.61 ± 0.04	1.98 ± 0.02	11.60 ± 1.70	Placebo
D	6.46 ± 0.30	1.88 ± 0.05	1.96 ± 0.03	5.75 ± 2.20	Placebo

SD: Standard deviation, *n* = 20.
